# Population genomics shed light on the demographic and adaptive histories of European invasion in the Pacific oyster, *Crassostrea gigas*

**DOI:** 10.1111/eva.12086

**Published:** 2013-07-24

**Authors:** Audrey Rohfritsch, Nicolas Bierne, Pierre Boudry, Serge Heurtebise, Florence Cornette, Sylvie Lapègue

**Affiliations:** 1 Ifremer, Laboratoire de génétique et pathologie des mollusques marinsLa Tremblade, France; 2 Département Biologie Intégrative, ISEMSète, France; 3 Ifremer, Laboratoire des Sciences de l'Environnement MarinPlouzané, France

**Keywords:** AFLPs, *Crassostrea gigas*, genome scan, invasive species, microsatellites, SNPs

## Abstract

*Crassostrea gigas* originated from the Pacific coast of Asia, but was introduced into several European countries in the early 1970s. Natural populations have now spread across the length of the western seaboard of Europe. To elucidate the demographic and selective processes at play during this rapid expansion, genome-scan analysis was performed on different populations. High diversities and low differentiation were observed overall, but significant genetic differentiation was found among newly established populations and between the newly established northern group and a nearly panmictic group composed of southern European populations and a population from Japan. Loss of genetic diversity was also seen in the north, likely caused by founder events during colonization. The few strongly supported outlier loci revealed a genetic structure uncorrelated with the north/south differentiation, but grouping two samples from the Danish fjords (northern group) and one from the Dutch Scheldt estuary (southern group) with the one from Japan. These findings might reflect the following: (i) parallel adaptation to similar environmental pressures (fjord-like environment) within each of the two groups or (ii) a footprint of a secondary introduction of an alternative genomic background maintained by multifarious isolation factors. Our results call for a closer examination of adaptive genetic structure in the area of origin.

## Introduction

During the last century, many species were intentionally translocated beyond their natural geographical ranges for commercial reasons, including cultivation. Some of these species were able to spread autonomously and have caused economic and/or environmental damage (Keller and Lodge 2007), that is, they became invasive. The Pacific oyster, *Crassostrea gigas*, is endemic to the Pacific coast of Asia, but has been translocated and introduced into many countries worldwide, mainly for aquaculture purposes (CIESM 2000). *C. gigas* was massively introduced into France at the beginning of the 1970s (Grizel and Héral 1991) and propagated in northern European countries by hatchery-based seed production (Troost 2010). For more than 20 years, *C. gigas* was only reproductive in France. The species failed to settle in northern Europe until the 1990s, when populations were reported to extend as far north as Norway. This expansion may have been the result of (i) natural dispersion, (ii) an increased ability of transplanted stocks to reproduce locally or (iii) a combination of both these processes. Hence, from the 1990s, the area of reproduction started to expand. Pacific oysters began to reproduce in Brittany (France) and now form large reefs in soft sediments and on rocks in this area (Lejart and Hily 2011). A northward range expansion was then observed in the Netherlands (Nehring 2006), throughout the Wadden Sea (Bruins 1983; Reise 1998; Nehring 1999; Dankers et al. 2004), and as far north as Sweden, where dense populations of recently settled oyster populations have been observed in several shallow-water sites (Strand et al. 2012). In Norway, populations can now be found in the shallow waters of the southern and western coasts (Wrange et al. 2010).

The colonization of these regions could have resulted from transplantation. Hence, in France, as soon as the Pacific oyster began to establish in southern bays, large spat and stock exchanges were started to enhance cultivation in both southern and northern areas of the Atlantic coast and were also made between the Atlantic Ocean and the Mediterranean Sea (Grizel and Héral 1991). In Scandinavia, attempts to cultivate oysters were made in Limfjorden, Denmark (Jensen and Knudsen 2005), in Sweden (oyster spat imported from Wales between 1973 and 1976; Eklund et al. 1977) and in Norway (since 1979; Strand and Vollstad 1997). Trials indicated that the environmental conditions were suitable for cultivation, but reproduction was not reported. In the 1980s, farmers imported Pacific oyster seed from Scotland to Norway. After strict restrictions were put on the importation of molluscs for cultivation purposes, local hatcheries started to produce their own spat in there from 1987 to 1990 (Strand and Vollstad 1997). Consequently, some of these animals may have founded local populations when environmental conditions became favourable, which then formed the basis for regional expansion. Indeed, climate modifications over the last 40 years may have allowed the species to reproduce in more areas. Hence, warm summers and mild winters may have contributed to the settlement of the species in some northern areas (Troost 2010). Northern European fjords could represent a favourable environment for this phenomenon, allowing them to maintain a sufficient adult population size, as the tidal forces are negligible in these areas and the oysters would be less exposed to conditions of extreme cold. Furthermore, the Pacific oyster is known to have a very wide range of tolerance to several factors, such as temperature, salinity and pathogens, with a high competitive ability that contributes to its invasive success. Hence, the temperatures at which the species can survive range from sub-zero (Quayle 1969; Diederich et al. 2005) to approximately +30°C (Le Gall and Raillard 1988; Bougrier et al. 1995). Furthermore, this species was successfully introduced into Europe and appeared tolerant to the iridovirus associated with the decline of the Portuguese oyster, *Crassostrea angulata* (Grizel and Héral 1991). Lastly, *Crassostrea gigas* was shown to have a high production yield in the natural environment in France (His 1972; Héral et al. 1986), and double the growth rate observed for the Portuguese oyster (Bougrier et al. 1986).

In this context, one might ask whether the success of this species, especially the northward expansion of its geographical range, could be explained solely by the opportunity to colonize an empty niche, in which case the spread would have depended mainly on demographic processes, possibly facilitated by global warming, or whether local adaptation was also necessary. A genome scan of differentiation (Lewontin and Krakauer 1973; Beaumont and Nichols 1996) between the area of origin, site of introduction and newly colonized sites might be one approach to conjointly identify loci affected by selection and examine demographic effects during introduction and colonization using a subset of markers that do not deviate from neutral expectations. Furthermore, outlier loci might under some circumstances prove useful to characterize repeated introductions that might not be visible with neutral markers if random genetic drift is not strong enough or if high propagule pressure and recurrent introduction from several sources have brought the full diversity of the native stock (Facon et al. 2008; Estoup and Guillemaud 2010; Riquet et al. 2013). Numerous examples exist showing that when population differentiation is found to be higher than the observed genomic average, this can be attributed to locally variable selection (Beaumont 2005; Nosil et al. 2009). However, alternative scenarios might also produce *F*_ST_ outliers (Excoffier and Ray 2008; Bierne et al. 2011, 2013b). Among such scenarios, the gene-surfing effect produced in the wave front of an expanding population (Excoffier et al. 2009b; Hofer et al. 2009) needs to be seriously considered when studying invasive species. Neutral allele surfing, like selection, would also occur at just a few loci and would therefore not affect all loci uniformly, unlike other demographic factors such as demographic expansions, inbreeding or bottlenecks. Furthermore, there have only been ∼40 generations since *C. gigas* was first introduced into Europe. This seems a short time lag for adaptation to have arisen from new mutations (i.e. the best situation to detect the signature of selection), although high fecundities and the large size of oyster populations would allow an appreciable influx of new mutations in each generation. During invasion, local adaptation is likely to produce so-called soft sweeps (Pennings and Hermisson 2006) because it should proceed from standing genetic variation through small allele frequency changes at many loci and would, thus, not result in a large *F*_ST_ at any locus (Le Corre and Kremer 2003, 2012). Shifts in allele frequency are thus expected only to be visible for strong selection coefficients and with markers very closely linked to selected loci or directly affected by selection.

However, the short time lag of invasion does not necessarily hold back adaptation if multiple introductions occur (Facon et al. 2006). Multiple introductions can introduce genotypes adapted to different environments, or partially isolated genomic backgrounds that have had sufficient time to diverge in the native range via multifarious evolutionary processes, which may replicate the adaptive differentiation in the invaded range. The mixing of differentiated genotypes by multiple introductions is thought to be an important process in adaptation during invasion (Facon et al. 2008; Estoup and Guillemaud 2010).

To date, very few studies of invasive species have used genome scan to investigate the potential role of adaptation during invasion (Prentis and Pavasovic 2013; Riquet et al. 2013). Interestingly, a recent scan of differentiation in an invasive marine invertebrate, the gastropod *Crepidula fornicata*, identified differentiated backgrounds characterized by a high rate of outliers in the native range of the species in America, but only one background was proved to have invaded Europe, and no outliers have been identified in the invaded range (Riquet et al. 2013). These authors suggested that genome scans with an insufficient marker density are unlikely to reveal adaptation during invasion from the introduction of a single background, even if adaptation has occurred, and advocated that other methods should be used in such cases (e.g. the analysis of phenotypic traits or gene expression, Mayrose et al. 2011; Hodgins et al. 2013).

The present article investigates whether selection has occurred in different European populations of *C. gigas*. Genetic analyses employed 8 microsatellites, 240 AFLPs and 30 SNP genetic markers. We used several methods to strengthen our detection of outliers and avoid the detection of false positives. These methods vary in stringency and have different potential biases. Lewontin and Krakauer (1973) developed the first test to disentangle the effects of neutral events from natural selection acting on particular loci, based on the distribution of *F*_ST_ across loci and the identification of outlier loci. In the approach of Beaumont and Nichols (1996), the distribution of *F*_ST_ across loci is plotted as a function of heterozygosity between populations, and neutral expectations are simulated under an infinite island model. Both methods rely on the island model of population structure, which can be a problem when the spatial structure generates correlation in co-ancestry (Nei and Maruyama 1975; Robertson 1975; Bonhomme et al. 2010; Bierne et al. 2013b; Fourcade et al. 2013). Excoffier et al. (2009a) modified the method of Beaumont and Nichols (1996) by introducing a hierarchical island model to perform simulations and account for correlation in co-ancestry within groups of a hierarchical structure of populations. Furthermore, Bonhomme et al. (2010) attempted to minimize false positives by accounting for more complex demographic structures using a statistic based on Lewontin and Krakauer's TLK and added phylogenetic estimation of the population's kinship matrix to account for historical branching. Foll and Gaggiotti (2008) proposed a Bayesian approach in which allele frequencies within populations are assumed to follow a multinomial Dirichlet distribution. In their method, *F*_ST_ is broken down into a population-specific component shared by all loci, and a locus-specific component is shared by all populations. Departure from neutrality at a given locus is assumed when the locus-specific component is necessary to explain the observed pattern of diversity. In this model, sampled populations are allowed to receive unequal numbers of migrants from the migrant pool, but can still lead to biases if migrant genes did not originate from the same pool (Excoffier et al. 2009a; Fourcade et al. 2013). Taking into account the different potential biases of those methods, we took a cautious conservative approach by combining the results of several methods, as recommended by Pérez-Figueroa et al. (2010).

In the present article, we report the study of *C. gigas* populations sampled across Europe and in the native range of the species using a variety of molecular markers and aim to document their genetic variability and population structure and investigate their adaptive response using a genome-scan approach.

## Materials and methods

### Sampling and marker amplification

**Figure 1:**
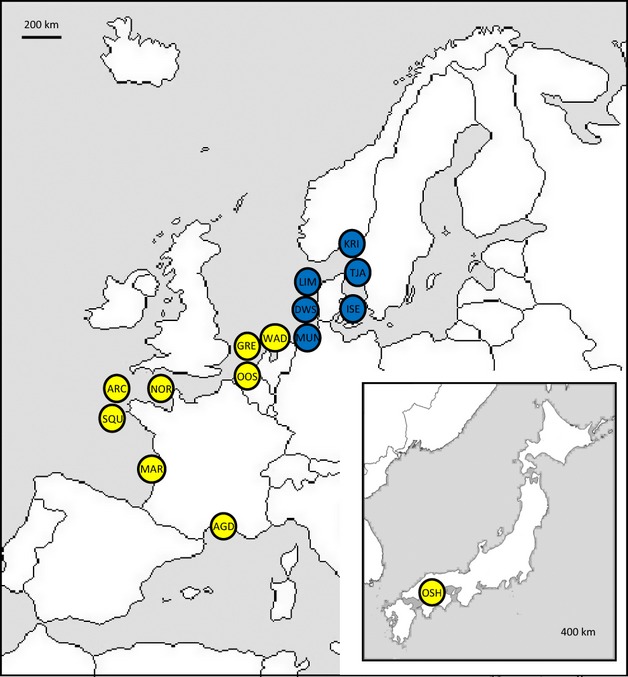
Sampling locations of *Crassostrea gigas* in Europe and Japan. AGD, Agde; ARC, Arcouest; DWS, Danish Wadden Sea; GRE, Grevelingen; ISE, Isefjord; KRI, Kristinberg; LIM, Limfjord; MAR, Marennes; MUN, Munkmarsch; NOR, Normandie; OOS, Oosterschelde; OSH, Iroshima; QUI, Quiberon; SQU, Squiffiec; TJA, Tjarno; WAD, Dutch Wadden Sea. The two colours represent the two different groups to which the populations belong according to the correspondence analysis and clustering analysis.

For each of the 746 individuals sampled across 16 populations (Table 1, Fig. 1), DNA was extracted from gill tissue using a chloroform extraction followed by purification with the Wizard(R) DNA Clean-Up System (Promega, Madison, WI, USA), according to Wilding et al. (2001). Concentrations were adjusted to 100 ng/μL.

**Table 1:** Sample characteristics and analysis effort

Population	Country	Label	Sampling size	AFLPs	SNPs	Microsatellites	Sampling date
Oshima	Japan	OSH	48	X	X	X	2008
Cap d'Agde	France	AGD	46	X	X	X	2008
Marennes	France	MAR	48	X	X	X	2008
Quiberon	France	QUI	48		X	X	2005
Squiffiec	France	SQU	48	X	X	X	2005
Arcouest	France	ARC	48		X	X	2005
Normandie	France	NOR	42		X	X	2008
Oosterschelde	The Netherlands	OOS	48	X	X	X	2006
Grevelingen	The Netherlands	GRE	46		X	X	2006
Dutch Wadden Sea	The Netherlands	WAD	48	X	X	X	2006
Munkmarsch	Germany	MUN	48	X	X	X	2006
Danish Wadden Sea	Denmark	DWS	48	X	X	X	2008
Limfjord	Denmark	LIM	48	X	X	X	2008
Isefjord	Denmark	ISE	36	X	X	X	2008
Tjarno	Sweden	TJA	48	X	X	X	2007
Kristenberg	Sweden	KRI	48	X	X	X	2007

The 30 single nucleotide polymorphism markers (Table 2) were developed as described in Sauvage et al. (2007) and genotyped using MALDI-TOF mass spectrometry (matrix-assisted laser desorption/ionization time of flight, Griffin and Smith 2000) at the Max Planck Institute genotyping platform in Berlin (Germany). Briefly, primers were designed using Primer 3 (Rozen and Skaletsky 2000) in ESTs of interest related to summer mortality, identified through a suppression subtractive hybridization approach (Huvet et al. 2004a), or were selected from existing databases such as GigasBase (http://public-contigbrowser.sigenae.org:9090/Crassostrea_gigas/index.html, Fleury et al. 2009) and Genbank.

**Table 2:** List of single nucleotide polymorphisms studied

Number	Usual and full names	Accession Number	Reference
1	**ATPase H+** *C. gigas* ATPase H+ transporting lysosomal protein gene, partial cds	EF694094	Sauvage et al. (2007)
2	**Glutaryl-CoA dehydrogenase–like protein** *C. gigas* glutaryl-CoA dehydrogenase–like protein gene, partial cds	EF694089	Sauvage et al. (2007)
3	Amylase gene B *C. gigas* alpha amylase (amy) gene, amy-B1 allele, partial cds	EF682217	Sauvage et al. (2007)
4	Gonadal Transforming Growth factor (ogTGFb) cDN21 *Crassostrea gigas* cDNA clone cDN21P0003A17 5-, mRNA sequence	AM856765	Fleury et al. (2009)
5	NADH dehydrogenase 6 EST016 CgG2RSMorest *C. gigas* cDNA clone HA26 similar to NADH dehydrogenase 6, mRNA sequence	CK172316	Huvet et al. (2004a)
6	Glucose 6 phosphatase *C. gigas* glucose-6-phosphatase gene, partial cds	EF694098	Sauvage et al. (2007)
7	**Glycogen synthase** *C. gigas* glycogen synthase gene, partial sequence	EF694079	Sauvage et al. (2007)
8	HA114_1 *C. gigas* clone HA114 genomic sequence	EF694082	Sauvage et al. (2007)
9	Ik cytokine-down regulator of HLA_2 *C. gigas* IK cytokine gene, partial cds	EF999946	Sauvage et al. (2007)
10	Laccase_1 *C. gigas* laccase gene, partial cds	EF999948	Sauvage et al. (2007)
11	Notch *C. gigas* notch3-like protein gene, partial cds	EF999949	Sauvage et al. (2007)
12	Superoxide dismutase *C. gigas* superoxide dismutase gene, partial cds	EF694097	Sauvage et al. (2007)
13	RNA helicase *C. gigas* partial mRNA for RNA helicase	AJ557014	A. Herpin (unpublished)
14	Amylase gene A *C. gigas* alpha amylase (amy) gene, amy-A1 allele, partial cds	EF694074	Sauvage et al. (2007)
15	Astacin *C. gigas* astacin metalloprotease gene, partial cds	EF694085	Sauvage et al. (2007)
16	Ferritin GF2 *C. gigas* ferritin GF2 (GF2) mRNA, complete cds	AY321300	Gueguen et al. (2003)
17	Bcl-2*C. gigas* predicted Bcl-2 protein mRNA	EU678310	Renault et al. (2011)
18	BQ426586 *C. gigas* Hemocytes Lambda Zap Express Library *Crassostrea gigas* cDNA, mRNA sequence	BQ426586	Gueguen et al. (2003)
19	**BQ427367** *C. gigas* putative immune protein gene, partial cds	EF694095	Sauvage et al. (2007)
20	Calcium dependant protein kinase *C. gigas* calcium-dependent protein kinase-like gene, partial sequence	EF694100	Sauvage et al. (2007)
21	**Glutathione S-transferase** *C. gigas* glutathione S-transferase sigma class gene, partial cds	EF694090	Sauvage et al. (2007)
22	Drac 3 *C. gigas* Drac3-like protein gene, partial cds	EF694083	Sauvage et al. (2007)
23	Flavin-containing mono oxygenase 2 *C. gigas* mRNA for flavin-containing monooxygenase 2 (fmo-2 gene)	AJ585074	Boutet et al. (2004)
24	BQ426639 *C. gigas* Hemocytes Lambda Zap Express Library *Crassostrea gigas* cDNA, mRNA sequence	BQ426639	Gueguen et al. (2003)
25	Glycoprotein hormone receptor *C. gigas* glycoprotein hormone receptor gene, partial cds	EF694088	Sauvage et al. (2007)
26	**HA114_2** *C. gigas* clone HA114 genomic sequence	EF694082	Sauvage et al. (2007)
27	Ik cytokine-down regulator of HLA_1 *C. gigas* IK cytokine gene, partial cds	EF999946	Sauvage et al. (2007)
28	Laccase_2 *C. gigas* laccase gene, partial cds	EF999948	Sauvage et al. (2007)
29	Sodium/glucose cotransporter_2 *C. gigas* sodium/glucose cotransporter mRNA, complete cds	AY551098	Huvet et al. (2004a)
30	Tubulin *C. gigas* tubulin gene, partial cds	EF694087	Sauvage et al. (2007)

Outlier loci are indicated in bold.

Microsatellite analysis was performed with 8 markers, according to the initial protocols for L48 (Huvet et al. 2000a), CGE09 (Yu and Li 2007), AMY (Sellos et al. 2003), sili29 and sili44 (Sauvage et al. 2009). A multiplex protocol was used for L10, CG49 and CG108 (Taris et al. 2005).

AFLP analyses for 4 primer pairs (Table S1) were performed using a modified version of Vos et al. (1995). For each sample, 250 ng of genomic DNA was digested with 1.25 units EcoRI (NEB) and MseI (NEB) and ligated with 100 units T4 DNA ligase (NEB), 0.1 μm EcoRI adapter and 1 μm MseI adapter [sequences in Vos et al. (1995)] in 25 μL 1X NEB buffer 2, 1X BSA and 1X manufacturer's ligase buffer for 16 h at 16°C. Preselective PCRs were performed on 2.5 μL diluted ligation (1:9 in 0 X TE) in 25 μL of 0.5 μm preselective primers, 0.2 mm of each dNTP, 1.5 mm MgCl_2_ and 0.5 units GoTaq (Promega) in manufacturer's buffer. Selective PCRs were performed on 5 μL diluted preselective amplification product (1:9 in 0 X TE) in 20 μL of 5 ng selective primers, 0.2 mm of each dNTP, 1.5 mm MgCl_2_ and 0.4 units GoTaq (Promega) in manufacturer's buffer. Conditions for preselective and selective PCRs were as given in Vos et al. (1995).

Electrophoresis and data collection were carried out on an ABI3100 sequencer (Applied Biosystems, Foster City, CA, USA) for both AFLP and microsatellite loci.

### Diversity and genetic structure

After automatic analysis with GeneMapper® software, AFLP genotypes were filtered using AFLPScore (Whitlock et al. 2008) to minimize error rate (<10%).

A between-class analysis, which is a specific type of correspondence analysis (CA) where each class is a population, was performed for all loci (AFLP, microsatellite and SNP) with ade4 package implemented in R (Chessel et al. 2004). The significance of observed structure was tested using a Monte Carlo test on the between-groups inertia percentage with 1000 randomizations.

Nonbiased heterozygosity (Hnb) (Nei 1978) and pairwise *F*_ST_ (1000 bootstraps) were computed using genetix 4.03 (Belkhir et al. 2001) for SNPs and microsatellites. Allelic richness was computed using FSTAT2.9.3 (Goudet 2001) for microsatellite loci.

For AFLPs, allele frequencies were first estimated using a Bayesian method with nonuniform prior distribution of allele frequencies (Zhivotovsky 1999). Expected heterozygosity under Hardy–Weinberg genotypic proportions (Hj) and pairwise *F*_ST_ were then computed using AFLP-SURV 1.0 (Vekemans 2002). Significance of observed differences in diversities between northern and southern populations was tested with Mann–Whitney–Wilcoxon tests (implemented in R 2.15; R Core Team 2012).

To infer the number of major genetic clusters, the Bayesian MCMC clustering approach was used, implemented in structure 2.3.1 (Pritchard et al. 2000). The admixture model with correlated allele frequencies was used to indicate the most likely pattern of population connectivity. Ten independent trials were run for each predefined number of clusters (*K*), with *K* = 1–10 and a burn-in of 50 000 iterations followed by 300 000 MCMC repetitions. We considered both raw probability values of ln*P*(*X*|*K*) and the Δ*K* estimate (Evanno et al. 2005). Codominant (microsatellites and SNPs) and dominant (AFLPs) markers were analysed separately.

### Outlier detection

For technical reasons related to the quality and quantity of DNA, AFLP genotyping was only possible on 12 of the 16 available samples. Detection of AFLP outlier loci was performed using a version of FDIST2 software (Beaumont and Nichols 1996) modified for dominant markers (DFDIST program; http://www.rubic.rdg.ac.uk/∼mab/stuff), Bayescan v2.0 (Foll and Gaggiotti 2008) and the method of Bonhomme et al. (2010). Frequencies of null alleles were computed using the approach of Zhivotovsky (1999). In DFDIST, a mean ‘neutral’ *F*_ST_ value was calculated after trimming 30% of the highest and lowest *F*_ST_ values (see Gagnaire et al. 2009). The number of demes was set at 100, and 50 000 loci were generated by coalescent simulations under the finite island model. The maximum frequency of the commonest allele allowed was set at 0.99. BayeScan was used with default parameters for the chain and model, with 50 000 iterations. Using the approach of Bonhomme et al. (2010), a matrix of Reynold's distances was computed in AFLP-SURV 1.0 (Vekemans 2002); a population sampled in Japan was used as the out-group and 50 000 iterations were made. The chi-square-approximated *P*-values were corrected for multiple testing according to the Benjamin–Benjamini-Hochberg method (as suggested in Bonhomme et al. 2010).

Detection of microsatellite and SNP outlier loci was performed using a version of DFDIST modified by r. Vitalis to simulate codominant, bi-allelic data (see Ségurel et al. 2010); the method of Excoffier et al. (2009a), implemented in Arlequin 3.5 (Excoffier and Lischer 2010); the method of Foll and Gaggiotti (2008), implemented in Bayescan v2.0; and the method of Bonhomme et al. (2010). For the last two methods, the parameters were the same as for the AFLPs. In R. Vitalis' modified version of DFDIST, 50 000 simulations were performed with 100 demes. The maximum frequency of the commonest allele allowed was set at 0.99. Because the finite island model has recently been shown to lead to a large fraction of false positives if populations are hierarchically subdivided, we used the modified version of FDIST implemented by Excoffier et al. (2009a) for codominant data that use a hierarchical island population model (as defined by Slatkin and Voelm 1991). Two groups of 100 demes were used (following the genetic structure results), that is, northern populations (Danish Wadden Sea, Isefjord, Kristenberg, Limfjord, Munkmarsch and Tjarno) and southern ones (Arcouest, Cap d'Agde, Dutch Wadden Sea, Grevelingen, Marennes, Normandie, Oosterschelde, Quiberon, Squiffiec and Japan). We used 50 000 coalescent simulations for the hierarchical model.

For all methods and types of markers, outlier detection was performed on all populations and on southern populations and northern populations separately. The threshold for outlier detection with BayeScan was set at FDR = 0.05 for both AFLP and SNP.

## Results

### Genetic diversity within populations

Genotypes at almost 500 AFLP loci were automatically generated for twelve populations using GeneMapper (R) software. Only 240 were used for further analyses (AFLPScore error rate between 2.3 and 9.8%, Table S1).

**Figure 2:**
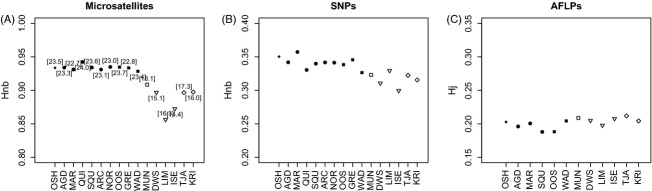
Plot of nonbiased expected heterozygosity (Hnb) and expected heterozygosity under Hardy–Weinberg genotypic proportions (Hj) from the different sample locations. White and black symbols indicate northern and southern populations, respectively. Mean allelic richness is shown in brackets for each population. A: Hnb for the 8 microsatellite loci, B: Hnb for the 30 SNPs, C: Hj for the 240 AFLPs.

Diversity estimators were plotted for the three kinds of markers (Fig. 2). Both microsatellite (Fig. 2A) and SNP (Fig. 2B) loci exhibited the same pattern of reduced diversity in populations from areas north of the Netherlands. For these northern samples (from Germany, Denmark and Sweden), nonbiased expected heterozygosities ranged from 0.8563 to 0.9085 and from 0.2996 to 0.3294 for microsatellites and SNPs, respectively. Mean allelic richness at microsatellite loci was between 14.4 and 18.1. For southern samples (from France to the Netherlands) and samples from Japan, heterozygosities were between 0.9284 and 0.9425 (mean allelic richness: 22.7 to 24.0) for microsatellite loci and between 0.3265 and 0.3573 for SNPs. For both microsatellites and SNPs, heterozygosities were significantly different between northern and southern populations (*W* = 0, *P*-value = 0.00025 and *W* = 1 *P*-value = 0.00050, respectively). For AFLPs (Fig. 2C), heterozygosities ranged from 0.1882 (Oosterschelde) to 0.2119 (Tjarno). No significant difference was observed between the two groups (*W* = 33, *P*-value = 0.99567).

### Population genetic structure

**Figure 3:**
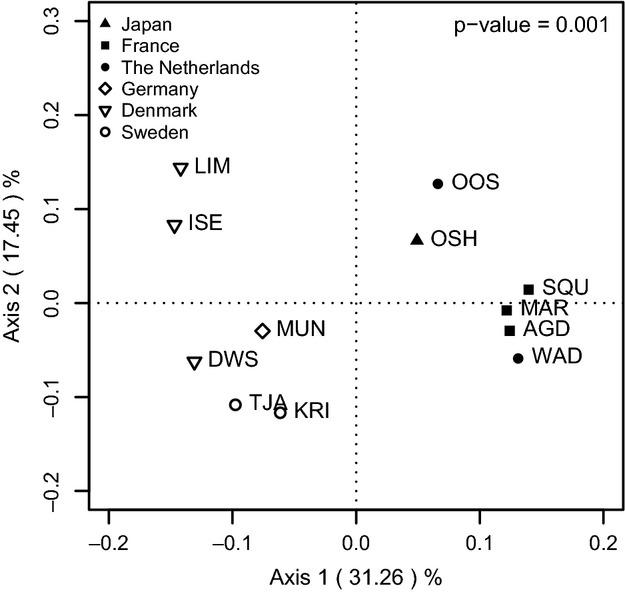
Projection of sample centres of gravity on the first factorial plane of a correspondence analysis (CA) on the matrix of allele frequencies at 278 loci (30 SNPs, 8 microsatellites and 240 AFLPs). White and black symbols indicate northern and southern populations, respectively.

For all types of loci (i.e. SNPs, microsatellites and AFLPs), global *F*_ST_ values were, respectively, 0.0129, 0.0160 and 0.0294, and all significantly different from zero (*P*-value > 0.001). Correspondence analysis on populations for all loci (Fig. 3) revealed two groups, which corresponded to the same ones identified on the basis of genetic diversity. The first of these groups included the ‘southern’ locations, with all populations from France, the Netherlands and Japan (endemic location). Global *F*_ST_ values were not significantly different from zero in this group (except for AFLPs when outliers were included). It is interesting to note that there is no genetic differentiation between the sample from Japan and any of those from the southern part of Europe. The second group encompasses the locations the furthest north in Europe, with populations located in Germany, Denmark and Sweden. In this group, global *F*_ST_ values were significantly different from zero (*P*-value < 0.001); the minimum value was found for microsatellites (0.0107) and the maximum for AFLPs (0.0243). The three populations from Denmark were significantly different from each other (Table S2).

Clustering analysis suggested a partition into two groups (*K* = 2) as statistically most likely for both codominant and dominant markers (Fig. S1: same colour code as in Fig. 1), thereby confirming the results of the correspondence analysis.

### Outlier detection

**Figure 4:**
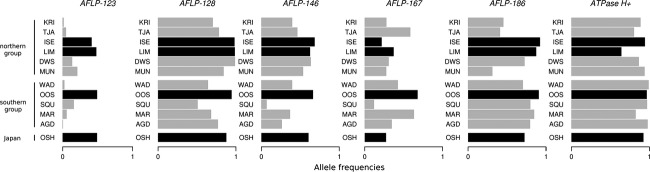
Plot of allele frequencies for the 5 AFLP outlier loci and ATPase H+ from the different locations.

**Figure 5:**
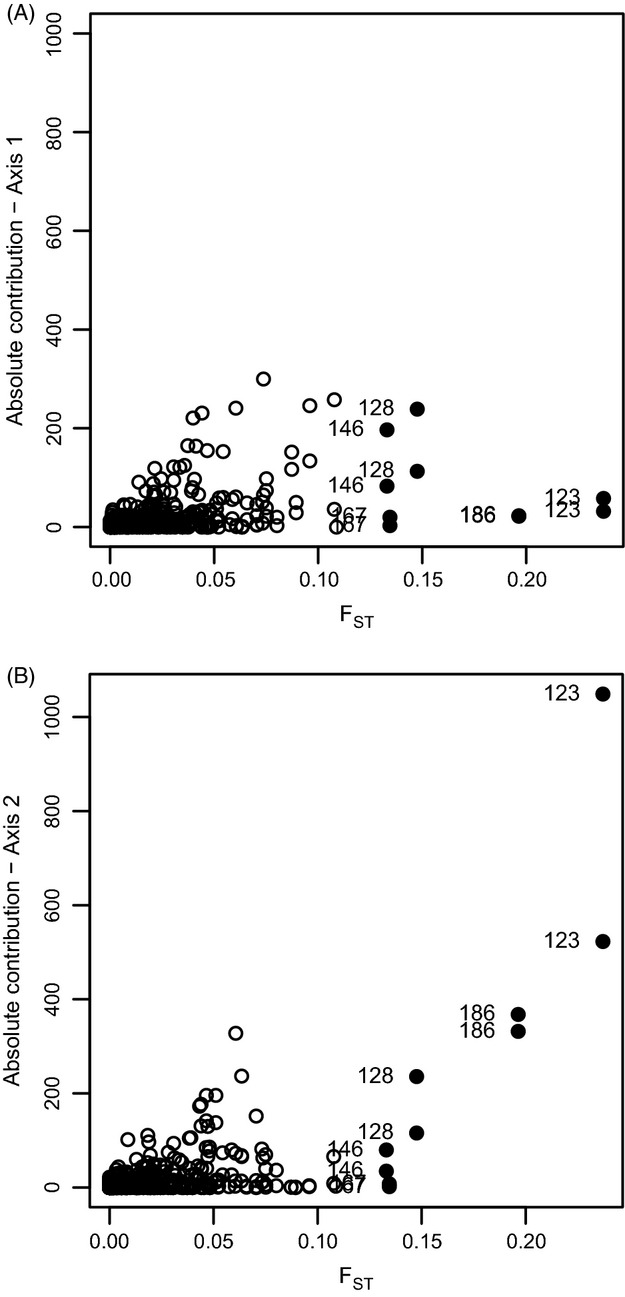
Absolute contributions of the decomposition of contribution for the alleles plotted against *F*_ST_ values for axes 1 (A) and 2 (B) for the 240 AFLP loci. The 5 outlier loci are plotted as black circles.

Results of outlier detection for all methods are presented in Table 3. We did not find any outliers with microsatellite markers. For AFLP loci, detection was performed on all populations using the approach of Bonhomme et al. (2010), and 23 loci were detected as outliers (corrected *P* value < 0.001). In northern and southern populations, five and eight loci were found to be outliers, respectively, by this method. With Bayescan, eight, three and five outliers were detected in the population groups ‘all’, ‘northern’ and ‘southern’, respectively. With DFDIST, seven, nine and eight outliers were found in all, northern and southern populations, respectively. For all populations, only four loci were identified as outliers with all methods (loci 123, 128, 146 and 186), only one (123) for northern populations and two (146 and 167) for southern ones. No particular geographical gradients in allele frequency were observed (Fig. 4), but four loci (123, 128, 146 and 186) tended to exhibit a similar structure. This is also visible on the second axis of the CA (Fig. 3), which is mainly explained by these four outliers (Fig. 5), and separates four samples with positive coordinates (ISE, LIM, OOS and OSH) from the others. Indeed, the correspondence analysis illustrates beautifully how outliers provide a pattern of genetic structure (axis 2) uncorrelated with the one observed with neutral markers (axis 1). Indeed, the outlier loci that contribute the most to axis 2 group together samples (OOS, OSH, ISE and LIM) that are otherwise differentiated from the other markers: OOS and OSH belonging to the southern group and ISE and LIM belonging to the northern group (which explains the north/south differentiation on axis 1). AFLP markers 123 and 186 in particular show a higher contributions to axis 2 (Fig. 5).

**Table 3:** Outlier detection for AFLP and SNP markers

Methods	AFLP/all	AFLP/North	AFLP/South	SNP/all	SNP/North	SNP/South
Bonhomme et al. (2010)*	19, 21, 23, 29, 55, 59, 71, 72, 76, 82, 94, 97, **123**, **128**, 137, **146**, 149, 160, 166, **186**, 187, 202, 219	19, 21, **123**, 154, 203	72, 82, 97, **146**, 149, 160, 166, **167**	ATPaseH+	None	None
BayeScan v2.0 (Foll and Gaggiotti 2008)†	96, **123**, **128**, 136, 141, **146**, 167, **186**	**123**, 128, 186	55, 123, 128, **146**, **167**	None	None	None
DFDIST (Beaumont and Nichols 1996)	88, **123**, **128**, 136, 167, **146**, **186**‡	19, 23, 29, **123**, 128, 137, 154, 186, 203‡	18, 123, 128, **146**, 160, **167**, 202, 223‡	None	None	None
DFDIST modified by Vitalis	NA	NA	NA	ATPaseH+, glycogen synthase, glutathione S-transferase, HA114_2‡	ATPaseH+, glutathione S-transferase ‡	Glutaryl-CoA, BQ427367 ‡
FDIST, hierarchical model (Excoffier et al. 2009a)	NA	NA	NA	ATPaseH+ ‡	NA	NA
Commun to all methods	**123, 128, 146, 186**	**123**	**146, 167**	ATPaseH+	NA	NA

* Locus with *P* value after Benjamini–Hochberg correction for multiple tests below 0.001.

† Locus detected as outliers with FDR=0.05.

‡ Locus with *P* value below 0.05.

NA, Not Applicable.

None: No outlier detected.

AFLP outlier loci indicated in bold are shared between all methods.

Such a pattern might have been suspected from the STRUCTURE analysis because OOS and LIM show a more similar pattern with AFLP markers (Fig. S1B), although the clustering analysis did not support a model with more than two clusters as the most likely.

For SNP loci, the method that detected most outliers was DFDIST. With this method, four loci were outside the envelope of neutral expectations for all populations (ATPase H+, glycogen synthase, glutathione S-transferase and HA114_2), two for northern ones (ATPase H+ and glutathione S-transferase) and two for southern ones (glutaryl-CoA dehydrogenase–like protein and BQ427367).

With the other methods, only ATPase H+ appeared to be a significant outlier for all populations. For this locus, allele frequency ranged from 0.6413 (Limfjord) to 0.9894 (Wadden Sea) with a mean of 0.902 (Fig. S1). The differentiation pattern observed at ATPase H+ was partly correlated with the one observed with AFLP outliers, and this locus also contributed substantially to axis 2 of the CA; however, it mainly separated the Danish fjords ISE and LIM from all the other populations. It should be noted that the northern/southern groups were not modified when outlier loci were removed from the structure analysis.

## Discussion

### Genetic diversity

As previously observed by Huvet et al. (2000b), no loss of diversity was noted when comparing the endemic population from Japan with populations sampled in southern Europe. Following the introduction of non-native species, a reduction in genetic diversity could occur due to founder effects, but this has never been observed in any real cases in bivalves (see Dlugosch and Parker 2008 for review) probably because of their high recruitment rates and recurrent introductions from several sources (Simberloff 2009). In the case of the introduction of *C. gigas* into France, repeated introductions of hundreds of tons of adults from British Columbia and thousands of tons of spat from Japan were made over the 5 years following the first massive introduction of adults at the beginning of the 1970s (Grizel and Héral 1991). Interestingly, genetic diversities observed in northern populations were lower for both microsatellites and SNPs, although no clear difference was observed with AFLP markers. A recent study performed in the Wadden Sea with a single mitochondrial locus surprisingly showed the opposite trend (Moehler et al. 2011), with a higher haplotype diversity in northern populations, which was attributed to a secondary introduction of hatchery spat from British Columbia. The observed loss of diversity is likely a signature of genetic drift on northern European coasts, where populations are smaller and more isolated than in the south. Previous studies have therefore shown examples of how spatial expansion can induce the structuring of newly colonized areas into distinct sectors of low genetic diversity (Excoffier et al. 2009b).

### Genetic structure

Low levels of genetic differentiation were observed in our study, especially within each group (*F*_ST_ = 0.000^NS^, in the south and *F*_ST_ = 0.0111 in the north). Likewise, marine species with long planktonic larval development (2–4 weeks in *Crassostrea gigas* according to water temperature and food availability) often exhibit low levels of population genetic structure related to high levels of gene flow (Hellberg et al. 2002). However, some studies have shown small but significant genetic structure in such ‘high gene flow’ marine species (Addison et al. 2008). Our observation is in accordance with previous studies in *C. gigas* that indicated a lack of genetic structure for mitochondrial and microsatellite DNA (Huvet et al. 2000b) between samples originating from southern Europe and Japan.

Despite this apparently low level of genetic structure, two groups can be distinguished that have higher *F*_ST_ values between them than within each group. The group with southern localities includes areas where *C. gigas* is currently cultivated: *F*_ST_ values for this group are very low and mainly not significantly different from zero. Farmers' practices with this species commonly involve translocation of stocks. Oysters grown in one region may have come from different origins (hatcheries or natural recruitments), sometimes very distant ones. These practices increase gene flow and therefore reduce genetic structure. The lack of genetic differentiation between Japan and southern Europe and the absence of diversity loss in the latter suggest that the bottleneck related to introduction was not sufficient to affect the genetic diversity, either because the number of founders was sufficiently high or because there have been repeated introductions (large propagule pressure and several sources of introduction; Roman and Darling 2007).

We found the same separation into two groups as Moehler et al. (2011), although this previous study was limited to the Wadden Sea. On the basis of one mitochondrial marker, these authors found virtually no genetic differentiation in the entire southern range of the Wadden Sea or in the putative source from British Columbia they had sampled. Although we do not have such a sample of the putative source in our study, we have a Japanese sample, and this does not show any differentiation from either the southern range of Wadden Sea or the rest of the Southern range sampled here in France. Furthermore, knowing that most of the oysters imported into France at the beginning of the 1970s came from British Columbia and Japan (Grizel and Héral 1991), one may hypothesize that the southern part of Europe up to the southern edge of the Wadden Sea, our sample from Japan and part of the British Columbia stocks may form a group of undifferentiated populations. However, this result also clearly shows the interest of better characterizing the genetic diversity present in the native range, especially the adaptive diversity, and comparing this with the European pattern.

The northern group is characterized by higher estimated *F*_ST_ values and lower diversities than the southern ones. It includes localities where *C. gigas* is no longer cultivated. These populations are presumed to have a different origin from those of the southern group. In Sylt (at the border between Germany and Denmark), spat was primarily imported from British and Irish hatcheries (Reise 1998; Nehring 1999), and the species began to spread around 1991. In Denmark, a large amount of *C. gigas* seed oysters were imported from England, the Netherlands and France to different locations for aquaculture experiments (Nehring 2006). In Isefjord, commercial production took place between 1986 and 1999. When oyster farming stopped, some oysters were left at the site, where they survived, but their numbers did not expand (Nehring 2006). The first free-living specimens were observed in 1999, which dispersed from the northern German Wadden Sea by natural means (Reise et al. 2005). The number of oysters that were imported and contributed to present populations in the northern part of Europe is likely to be relatively low compared with those produced and translocated each year in France (about 130 000 tons/year were transported around France, whereas only 100 000–300 000 individual oysters were produced annually in Isefjord). It can therefore be hypothesized that these recently settled northern populations evolved isolated from the southern ones with little or no gene flow caused by human activities. Moreover, among the 16 pairwise *F*_ST_ estimates above 0.05, ten involved populations from Denmark. Large variance in reproductive success, as previously reported in this species (Li and Hedgecock 1998; Boudry et al. 2002), is likely to occur in these relatively isolated populations; this could further enhance genetic drift, decreasing genetic diversity as explained above and increasing the observed genetic differentiation.

### Detection of selection

Among the 30 SNPs studied, one was detected as an outlier (3.3%) with all methods used. For AFLPs, four outliers (2%) were detected within the 240 AFLP loci scored, which could appear low compared with that given in the literature for other species. A review by Nosil et al. (2009) on 18 relevant papers dealing with ‘genome scan and selection’ showed substantial, but not extreme, variation in the proportion of outliers, with a range from 0.4% to 24.5% and a mean of 8.5% among the loci analysed. For *Littorina saxatilis* (an intertidal snail), 5% of 306 AFLP loci were consistently characterized as outliers between ecotypes on three shores (Wilding et al. 2001; Grahame et al. 2006; Wood et al. 2008). For *Crassostrea virginica*, only 1.4% of outliers were detected with 215 AFLP markers, although only two populations were studied in this work (Murray and Hare 2006). Nosil et al. (2009) concluded that approximately 5–10% of the genome is strongly affected by divergent selection. However, studies on some other marine invertebrates did not detect any outliers, such as in the periwinkle *Echinolittorina hawaiiensis* (Tice and Carlon 2011) or, interestingly, in the gastropod *Crepidula fornicata*, which invaded Europe at the same time as with oysters (Riquet et al. 2013). The discrepancy between the different studies on the proportion of outliers might be explained by the variation in the number of populations and individuals analysed, the markers used, the methods used for estimating baseline neutral differentiation or the criteria used for determining outlier status. However, more conceptual explanations have been put forward, such as the spatial structure that some organisms have to cope with, for example, a long tree-like linear habitat (Fourcade et al. 2013), or the timescale at which adaptation could have proceeded (Riquet et al. 2013). Bierne et al. (2013b) emphasized that in any case, outlier tests are simply not designed to account for pervasive selection and that when too high a fraction of the genome is affected by selection, the theory of hybrid zones and genetic barriers should be considered, rather than basic local adaptation. Additionally, in the case of introductions, the time lag can appear too short at first sight to suggest the existence of a genome-wide multifactorial, semipermeable genetic barrier to gene flow, but multiple introductions could well have imported differentiated backgrounds from the area of origin to the invaded zone. In the case of oysters, we already have one such example, as *C. angulata* was first introduced into Europe several centuries ago and *C. gigas* was introduced 40 years ago. The two species now hybridize in southern Portugal (Huvet et al. 2004b). Even under a simple model of single introduction and local adaptation, discrepancies among different surveys may also reflect the intensity of divergent selection and/or the time since divergence has been acting between populations (Renaut et al. 2011). Hence, under the hypothesis of a single introduction and spread, selection would have occurred on European populations of *C. gigas* for about 40 generations. This is a very short period, allowing the detection of very few, if any, outliers, as also recently observed in *Crepidula* (Riquet et al. 2013).

In the present article, we have presented a stringent detection of AFLP outliers, and our findings are in agreement with the results of a recent comparison between different methods showing that Bayescan usually detects a high percentage of true selective loci as well as <1% false-positive outliers (Pérez-Figueroa et al. 2010). Although the combination of the results of several methods could be considered a cautious approach, the same study highlighted that false positives are common even with a combination of methods and multitest correction, suggesting that the outliers detected should still be considered with extreme caution. Here, we were especially cautious about the north–south comparison for three reasons: (i) it generates a hierarchical structure (Excoffier et al. 2009a), (ii) the sampling was performed along a long linear habitat (Fourcade et al. 2013), and (iii) colonization can produce gene-surfing effects that might bias outlier tests available to date (Klopfstein et al. 2006; Excoffier et al. 2009b; Hofer et al. 2009). As the southern group proved to be panmictic, we repeated outlier tests in comparisons between each of the northern populations and the pooled data from the southern populations. Some outliers were detected in this way, but none were well supported by consistent repetition in multiple comparisons (Fig. S2). However, some detected outliers revealed a genetic structure that was independent of the differentiation between the northern and southern groups, as this latter genetic pattern was the same whether outlier loci were considered or not. These outliers that group populations from the southern and the northern groups are robust candidates for selection because they do not suffer from the problem of an increased variance of neutral *F*_ST_ due to shared co-ancestry (Robertson 1975; Excoffier et al. 2009a; Bonhomme et al. 2010; Bierne et al. 2013b).

Interestingly, among these most differentiated loci, we noticed a tendency for parallel genetic structure, grouping together some samples of the northern group in Denmark (ISE and LIM), a sample of the southern group in the Netherlands (OOS) and the Japanese sample (OSH). An uncorrelated structure between some outliers and the neutral fraction of the genome is clearly visible in the CA, in which the first axis reveals the genome-wide genetic structure between the southern and northern groups, while the second axis reveals the outlier-specific genetic structure that differentiates two samples of the southern group (OOS and OSH) and two samples of the northern group (ISE and LIM). The simplest hypothesis that could explain this pattern would be parallel adaptation to similar environmental pressures in these four populations, which is a hypothesis regularly put forward in the literature (Bradbury et al. 2010; Limborg et al. 2012). By parallel adaptation, we do not necessarily mean that there is independent primary differentiation caused by ecology-driven selection at different locations, but that adaptive polymorphisms associate more with the environment than with the neutral genetic structure (Bierne et al. 2013a). The fact that the Danish samples come from fjords and the Dutch sample from the Scheldt estuary in Zealand (the Netherlands) would point to adaptation to fjord-like environments. In addition to salinity, fjords and estuaries are characterized by multifarious abiotic (e.g. temperature and pH) and biotic (e.g. parasites, predators and competitors) ecological factors. However, we also noticed that each of the four samples individualized by outlier loci came from a site where cultivation has taken place during several periods over the last 40 years. We can therefore speculate that the history of introduction for aquaculture purposes could have interacted with selection to generate the genetic structure observed at outlier loci. Hence, the Danish fjords sampled (Limfjord and Isefjord), as well as the Oosterschelde population in the Netherlands, show higher frequencies at specific markers (Fig. 4), which may reflect the different origins of introduction that occurred in northern Europe and that are partly linked to aquaculture activity, as also concluded by Moehler et al. (2011). This hypothesis of a secondary introduction does not fully explain the uncorrelated effect of selection (outlier-specific) and neutral processes (genome-wide). To understand the genetic structure observed, we need to further assume that the genome of the hatchery stock would have been secondarily swamped by the wave of invasion at neutral markers, erasing the history of introduction at these markers, while the hatchery background would have been partially maintained at selected loci. Our results therefore call for a closer examination of the adaptive genetic structure in the area of the Pacific where this species originated, as well as in other areas where *C. gigas* is cultivated and that have served as a stepping stone before the introduction into Europe (e.g. British Columbia).

## Conclusion and perspectives

This genomic study on the invasiveness of the Pacific cupped oyster in Europe aimed to disentangle the demographic and adaptive factors as well as to consider the impact of aquaculture on the patterns of genetic structure observed. We used numerous markers for the analysis of a specific context of an economically important species during its invasive phase in Europe. We did not observe any loss of diversity during introduction, although some was seen during the northern spread of the species in Europe. There is little, if any, evidence of adaptation following the introduction of *Crassostrea gigas* into Europe, as there was no genetic differentiation or decrease in diversity between the population from Japan (the origin of European populations) and populations from areas of southern Europe where *C. gigas* is cultivated. However, northern populations (from Germany, Denmark and Sweden) showed a decrease in diversity, coupled with a stronger genetic structure. This result agrees with the assertions that (i) the number of oysters that participated in the introduction of *C. gigas* in northern populations was far lower than the large quantities introduced from Japan in the 1970s and (ii) that the invasion wave due to the acclimatization of the species in Europe has yet not reached northern latitudes, but is presently localized somewhere on the North Sea coast of Germany. From a practical point of view, this study demonstrates the genetic impact of aquaculture on a newly introduced species and consequently shows that care must be taken when considering the stocks that are chosen at the beginning of a breeding programme. Indeed, as there are very few means to manage diseases in molluscs (Renault 2011), breeding programmes, together with the modification of farmers' practices (exchanges, densities, etc.), appear to offer a solution to such problems and have begun to develop since 2011. Genetic characterization is clearly of interest for the choice of the initial broodstocks to be used, but also for the genetic diversity to be regularly monitored in such programmes.

## Data archiving statement

Data for this study are deposited in the Dryad repository: doi:10.5061/dryad.13jc4.

## Supporting Information

**Figure S1.** Bar plot of admixture proportions of individuals, estimated using STRUCTURE with K = 2 groups for microsatellite and SNP markers (A) and AFLP makers (B). Information on sample origin is given below each bar plot.

**Table S1.** Characteristics of the markers used for AFLP analysis.

**Table S2**. Estimates of Weir and Cockerham's (1984) θ between pairs of samples.
