# Supplementary figures and images for: Population genomics shed light on the demographic and adaptive histories of European invasion in the Pacific oyster, Crassostrea gigas
Source: Evol Appl. 2013 Jul 24;6(7):1064–78. doi: 10.1111/eva.12086 (PMC3804239; doi:10.1111/eva.12086)

**A**

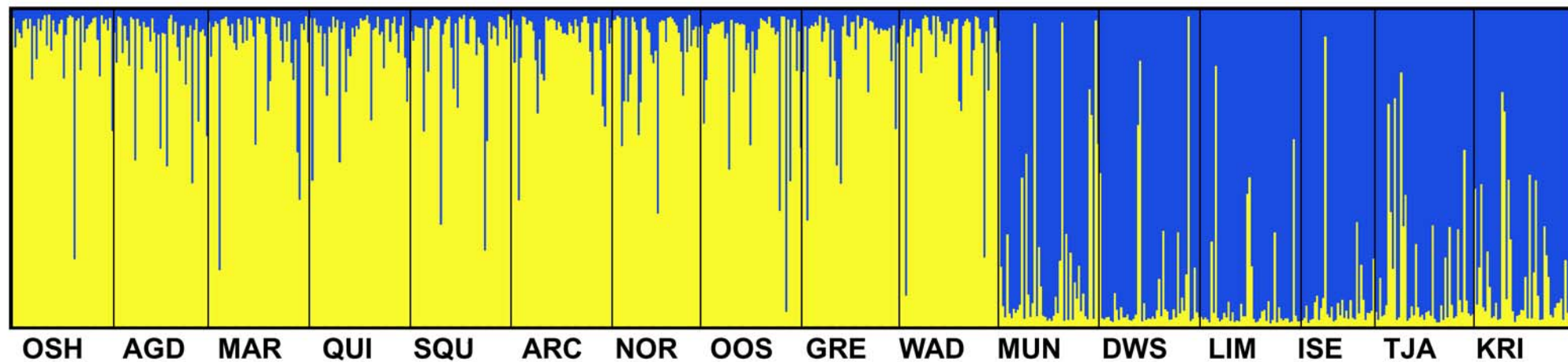

**B**

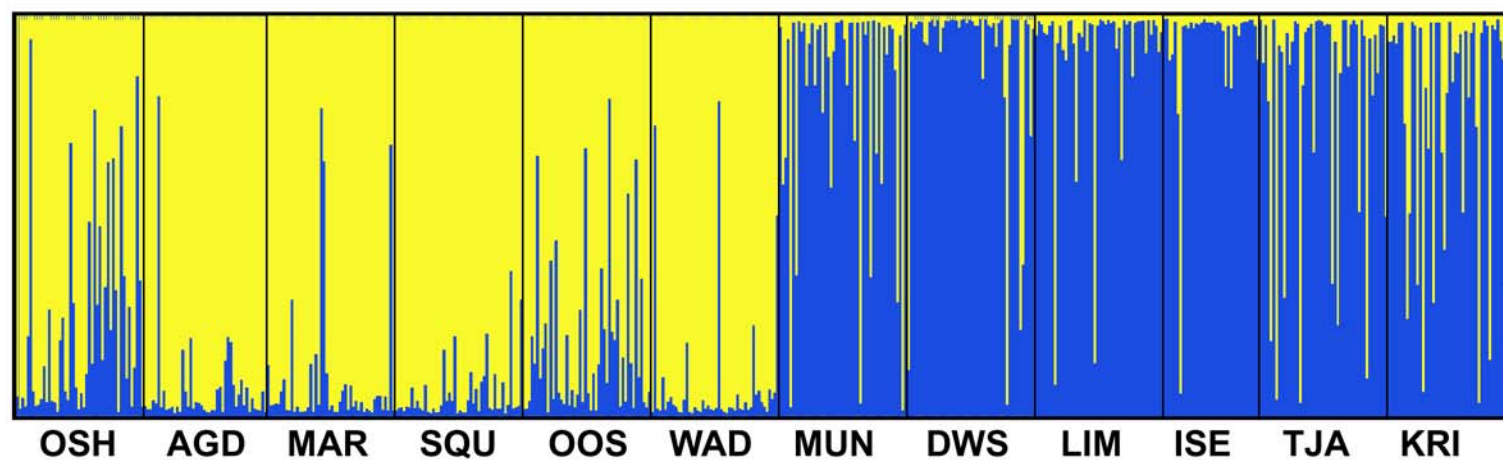

Supplement: Supplementary file 1 [file eva0006-1064-SD1.pdf]

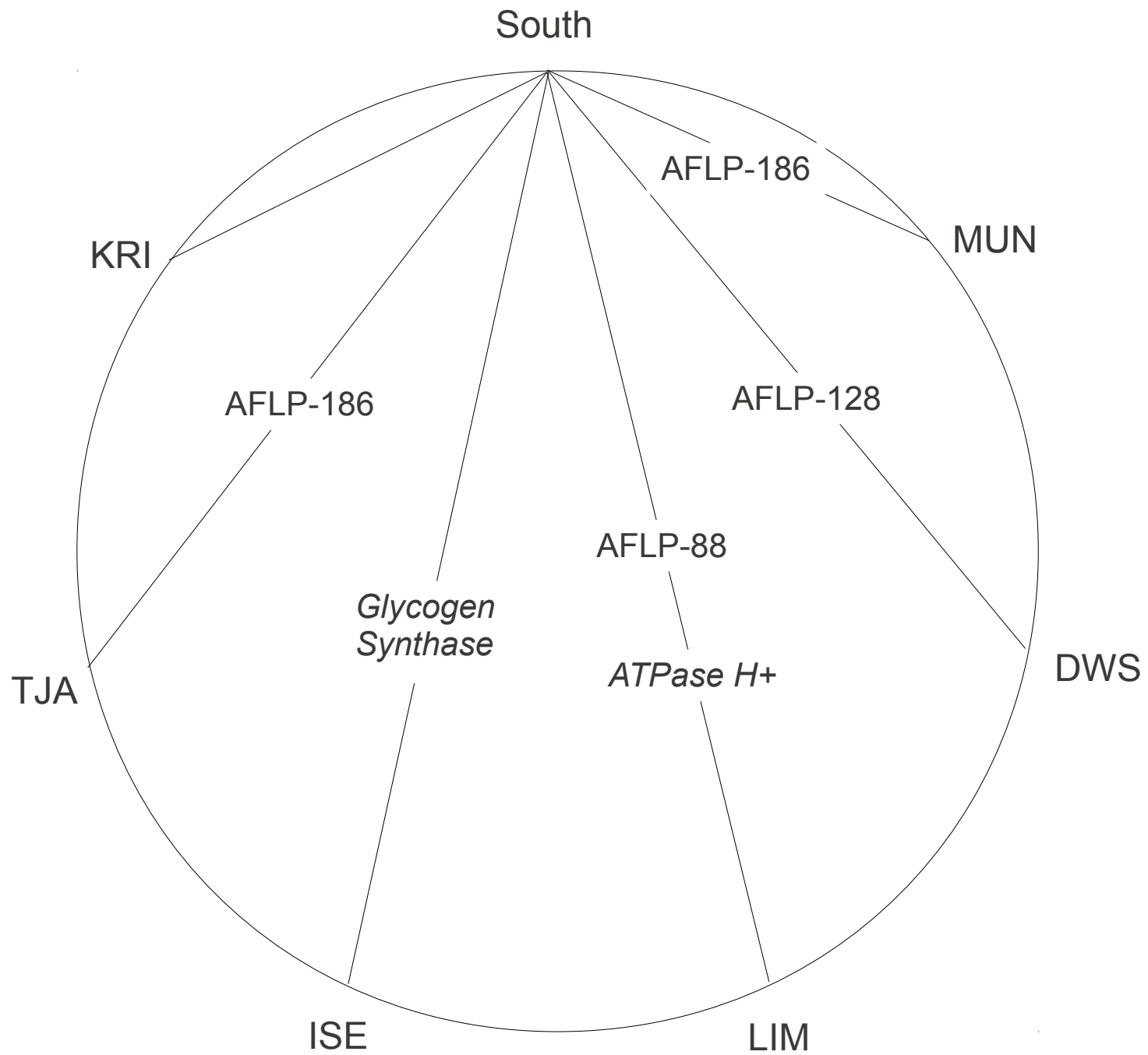

Supplement: Supplementary file 2 [file eva0006-1064-SD2.pdf]
