# Supplementary material for: Population genomics shed light on the demographic and adaptive histories of European invasion in the Pacific oyster, Crassostrea gigas
Source: Evol Appl. 2013 Jul 24;6(7):1064–78. doi: 10.1111/eva.12086 (PMC3804239; doi:10.1111/eva.12086)
Supplement: Supplementary file 3 [file eva0006-1064-SD3.docx]

**Supplementary materials**

**Table S1.** Characteristics of the markers used for AFLP analysis.

| Primer pair | Selective primers | Error rate | M | PLP (%) |
| --- | --- | --- | --- | --- |
| b7 | 5'-GACTGCGTACCAATTC**ACG**-3' EcoRI  5'-GATGAGTCCTGAGTAA**AGT**-3' MseI | 5.9 % | 15.9 | 53% |
| b10 | 5'-GACTGCGTACCAATTC**ACG**-3' EcoRI  5'-GATGAGTCCTGAGTAA**CAC**-3' MseI | 9.8 % | 8.9 | 64% |
| c7 | 5'-GACTGCGTACCAATTC**AAC**-3' EcoRI  5'-GATGAGTCCTGAGTAA**AGT**-3' MseI | 6.2 % | 26.0 | 91% |
| c10 | 5'-GACTGCGTACCAATTC**AAC**-3' EcoRI  5'-GATGAGTCCTGAGTAA**CAC**-3' MseI | 2.3 % | 14.6 | 40% |

M: Mean number of loci; PLP: percentage of polymorphic loci.

**Table S2**. Estimates of Weir and Cockerham’s (1984) θ between pairs of samples. Above diagonal: matrix for microsatellites; below diagonal: matrix for SNPs.

|  | OSH | AGD | MAR | QUI | SQU | ARC | NOR | OOS | GRE | WAD | MUN | DWS | LIM | ISE | TJA | KRI |
| --- | --- | --- | --- | --- | --- | --- | --- | --- | --- | --- | --- | --- | --- | --- | --- | --- |
| OSH |  | -0.00089 | 0.00084 | 0.00185 | 0.00146 | -0.00069 | 0.00319 | -0.00013 | 0.00561 | 0.00060 | **0.01934**  ******* | **0.02286**  ******* | **0.04251**  ******* | **0.03275**  ******* | **0.02538**  ******* | **0.02116**  ******* |
| AGD | -0.01321 |  | -0.00017 | 0.00070 | 0.00044 | -0.00216 | -0.00084 | -0.00359 | 0.00313 | -0.00456 | **0.01140**  ******* | **0.01396**  ******* | **0.03659**  ******* | **0.02427**  ******* | **0.02031**  ******* | **0.01382**  ******* |
| MAR | 0.00078 | 0.00785 |  | -0.00061 | -0.00063 | -0.00004 | 0.00164 | -0.00181 | -0.00143 | -0.00103 | **0.01179**  ******* | **0.02013**  ******* | **0.03744**  ******* | **0.02926**  ******* | **0.01913**  ******* | **0.01644**  ******* |
| QUI | -0.00354 | 0.00552 | 0.00202 |  | 0.00270 | -0.00060 | 0.00114 | -0.00158 | 0.00204 | 0.00036 | **0.01255**  ******* | **0.01960**  ******* | **0.03901**  ******* | **0.02670**  ******* | **0.01818**  ******* | **0.01592**  ******* |
| SQU | -0.00393 | 0.00352 | 0.00539 | 0.00327 |  | 0.00028 | 0.00086 | -0.00194 | -0.00037 | -0.00027 | **0.01374**  ******* | **0.01904**  ******* | **0.04054**  ******* | **0.03040**  ******* | **0.01803**  ******* | **0.01878**  ******* |
| ARC | 0.00306 | 0.00030 | -0.00322 | -0.00039 | -0.00028 |  | 0.00030 | -0.00359 | 0.00248 | -0.00245 | **0.01766**  ******* | **0.01891**  ******* | **0.04074**  ******* | **0.02550**  ******* | **0.02063**  ******* | **0.01633**  ******* |
| NOR | -0.01034 | 0.00117 | 0.00017 | 0.00130 | 0.01074 | -0.00189 |  | -0.00219 | 0.00291 | -0.00076 | **0.01400**  ******* | **0.01840**  ******* | **0.04241**  ******* | **0.02667**  ******* | **0.01904**  ******* | **0.01607**  ******* |
| OOS | 0.00050 | 0.00006 | 0.00996 | 0.00433 | 0.01144 | 0.00321 | 0.00205 |  | -0.00058 | -0.00356 | **0.00754**  ******* | **0.01241**  ******* | **0.03465**  ******* | **0.02379**  ******* | **0.01727**  ******* | **0.01063**  ******* |
| GRE | -0.01411 | -0.00476 | -0.00039 | -0.00165 | 0.00328 | -0.00291 | -0.00473 | -0.00400 |  | 0.00321 | **0.01324**  ******* | **0.01954**  ******* | **0.03949**  ******* | **0.03194**  ******* | **0.01976**  ******* | **0.01545**  ******* |
| WAD | -0.00764 | 0.00885 | 0.00187 | -0.00377 | 0.00504 | 0.00034 | 0.00102 | 0.00822 | 0.00086 |  | **0.01208**  ******* | **0.01598**  ******* | **0.03474**  ******* | **0.02630**  ******* | **0.01962**  ******* | **0.01680**  ******* |
| MUN | -0.00940 | 0.00446 | 0.01556 | 0.01068 | 0.01475 | 0.00616 | 0.01247 | 0.00605 | 0.00527 | 0.01170 |  | 0.00214 | **0.02006**  ******* | **0.01399**  ******* | 0.00855 | -0.00038 |
| DWS | 0.01499 | 0.01108 | **0.02252**  ******* | **0.02007**  ******* | **0.02269**  ******* | 0.01443 | **0.02183**  ******* | **0.01638**  ******* | 0.01306 | **0.02345**  ******* | 0.00083 |  | **0.01283**  ******* | 0.01103 | 0.00196 | 0.00024 |
| LIM | 0.03164 | **0.04690**  ******* | **0.04669**  ******* | **0.05050**  ******* | **0.04218**  ******* | **0.03693**  ******* | **0.04909**  ******* | **0.04906**  ******* | **0.04317**  ******* | **0.04714**  ******* | **0.01717**  ******* | 0.02962 |  | **0.03136**  ******* | **0.01917**  ******* | **0.01846**  ******* |
| ISE | 0.00900 | 0.01994 | **0.02950**  ******* | **0.03098**  ******* | **0.03229**  ******* | **0.02468**  ******* | 0.01819 | **0.03502**  ******* | **0.01998**  ******* | **0.03379**  ******* | 0.01278 | 0.02582 | 0.03822 |  | **0.01525**  ******* | **0.01276**  ******* |
| TJA | 0.01651 | 0.01241 | **0.02038**  ******* | **0.01833**  ******* | 0.01831 | 0.01457 | 0.01582 | **0.02411**  ******* | 0.01197 | **0.02131**  ******* | 0.00184 | -0.00058 | **0.01930**  ******* | 0.01094 |  | 0.00341 |
| KRI | 0.01142 | 0.01182 | **0.01889**  ******* | 0.01142 | 0.01512 | 0.00837 | 0.01469 | **0.01902**  ******* | 0.00946 | **0.01768**  ******* | -0.00164 | -0.00043 | 0.02102 | 0.00734 | -0.00383 |  |

***P<0.001 (1000 permutations under GENETIX (Belkhir
